# Understanding Driving Avoidance Among Older African Americans And Whites With Diabetes

**DOI:** 10.1093/geroni/igab046.2779

**Published:** 2021-12-17

**Authors:** Henrietta Armah, Maya Martin, Wesley Browning, Ghislaine Atkins, Olivio Clay

**Affiliations:** 1 University of Alabama at Birmingham, Birmingham, Alabama, United States; 2 Tuskegee University, Tuskegee, Alabama, United States

## Abstract

Diabetes mellitus is one of the most common chronic diseases with half of the new diagnoses affecting adults aged 60 years and older. Although African Americans are more likely to develop the disease, they are also less likely to receive healthcare. Importantly, living with diabetes is likely to negatively impact mobility for aging adults as the disease is associated with lower physical functioning (e.g., ability to maintain one’s balance). Further, diabetes could pose a significant threat to a person with diabetes’ ability to drive and remain in the community. This study examines the relationships and influences of social determinants of health (e.g., race, gender, socioeconomic status) and cognition on avoiding driving maneuvers such as driving at night and in rush hour traffic among older adults with diabetes. Data from the University of Alabama at Birmingham (UAB) Diabetes and Aging Study of Health (DASH) were analyzed and of the 224 participants, 193 (86.16%) were current drivers. There was a gender difference with 94.12% of males and 79.51% of females being current drivers, p < .01. Within the sample of current drivers, 45% were African American and being female, not married, lower levels of education and cognition, low income, and being African American were associated with higher scores on driving avoidance. Cognition explained 30.44% of the racial difference in driving avoidance. Findings from this study will help identify individuals who are at-risk for reduced mobility and identify those who may need to be intervened upon to support a better quality of life.

